# Rapid interferon independent expression of IFITM3 following T cell activation protects cells from influenza virus infection

**DOI:** 10.1371/journal.pone.0210132

**Published:** 2019-01-16

**Authors:** James G. Bedford, Meredith O’Keeffe, Patrick C. Reading, Linda M. Wakim

**Affiliations:** 1 Department of Microbiology and Immunology, The University of Melbourne, at the Peter Doherty Institute for Infection and Immunity, Melbourne, Victoria, Australia; 2 Infection and Immunity Program, Monash Biomedicine Discovery Institute and Department of Biochemistry and Molecular Biology, Monash University, Melbourne, Victoria, Australia; 3 WHO Collaborating Centre for Reference and Research on Influenza, Victorian Infectious Diseases Reference Laboratory, at the Peter Doherty Institute for Infection and Immunity, Melbourne, Victoria, Australia; University of Hong Kong, HONG KONG

## Abstract

Interferon-induced transmembrane protein 3 (IFITM3) is a potent antiviral protein that enhances cellular resistance to a variety of pathogens, including influenza virus. Classically defined as an interferon-stimulated gene, expression of IFITM3 on cells is rapidly up-regulated in response to type I and II interferon. Here we found that IFITM3 is rapidly up-regulated by T cells following their activation and this occurred independently of type I and II interferon and the interferon regulatory factors 3 and 7. Up-regulation of IFITM3 on effector T cells protected these cells from virus infection and imparted a survival advantage at sites of virus infection. Our results show that IFITM3 expression on effector T cells is crucial for these cells to mediate their effector function and highlights an interferon independent pathway for the induction of IFITM3 which, if targeted, could be an effective approach to harness the activity of IFITM3 for infection prevention.

## Introduction

Cells are equipped with a variety of mechanisms to protect themselves from virus infection. The early detection of a viral infection by innate receptors triggers the production of type I interferon (IFN), which in turn signals expression of interferon-stimulated genes (ISG) within the host cell. The proteins encoded by these genes interfere with viral replication and enhance the ability of uninfected cells to resist infection. Interferon-induced transmembrane 3 (IFITM3) is a potent anti-viral protein that exhibits protection against a broad range of viruses including orthomyxoviruses, flaviviruses, filoviruses, and coronaviruses [1–3]. IFITM3 is particularly effective at protecting against influenza virus infection and the absence of this single antiviral protein is associated with exacerbated influenza infection in both mice and humans [1, 4, 5]. As such, IFITM3 knockout mice succumb to sublethal doses of influenza virus [3, 6] and humans expressing a functionally defective IFITM3 allelic variant are more prone to severe influenza virus infection [7–10]. IFITM3 inhibits viral entry, the earliest step of the virus life cycle, by preventing viruses from traversing the lipid bilayer of the cell and accessing the cytoplasm [11]. IFITM3 is positioned in the lipid membranes of endosomes and lysosomes [12, 13] and traps endocytosed virus particles within these vesicles by interfering with the formation of the virus fusion pore [14, 15]. As a result, viruses that require pH-dependent triggering of viral fusion machinery to escape from the endosome into the cytosol are highly susceptible to the antiviral activities of IFTIM3 [1, 11, 16].

While some cell types, including respiratory epithelial cells [6] and tissue resident memory T cells [17] constitutively express IFITM3, many others do not express this antiviral protein in steady-state conditions [18] and only upregulate expression after exposure to type I or type II interferons [6, 19]. Expression levels of IFITM3 are tightly regulated by the E3 ubiquitin ligase, NEDD4, which reduces baseline levels of IFITM3 by ubiquitinating the antiviral protein, thereby promoting its turnover [20–22].

Whether IFITM3 expression is regulated differently in distinct cell types remains unclear. As immune cells often carry out effector functions at sites of infection and are therefore directly exposed to virus-infected cells, these cells are likely to benefit greatly from regulated expression of antiviral proteins. We have previously reported that dendritic cells stationed within the lung upregulate IFITM3 following influenza virus infection, a process driven by exposure to type I interferon, and this was crucial for these cells to successfully traffic influenza viral antigen from the lung to draining lymph node (LN) without becoming infected and perishing en route [23]. Tissue resident memory T cells in the airways [10], lung [17], skin [24] and brain [25] constitutively express IFITM3, and this is associated with enhanced resistance to viral infection and therefore long-term survival within peripheral tissues.

Here we extend this research to investigate the expression pattern and drivers of IFITM3 expression in T cells during their activation and infiltration into influenza virus-infected lung tissue. We find that IFITM3 is rapidly up-regulated on T cells following their activation in the draining LN and expression is maintained as these cells infiltrate sites of virus infection. Up-regulation of IFITM3 on effector T cells protected these cells from virus infection and imparted a survival advantage at sites of virus infection. Interestingly, the expression of IFITM3 on activated T cells was not driven by type I or II interferon and did not rely on the interferon regulatory factors 3 and 7. Our results show that IFITM3 expression on effector T cells is crucial for these cells to mediate their effector function and highlights an interferon independent pathway for the induction of IFITM3, which if targeted, could be an approach to harness the activity of IFITM3 for infection prevention.

## Material and methods

### Mice and viruses

C57BL/6, OT-I.CD45.1, IFITM3 KO (ifitm3^tm1(RFP)Zhang^, The Jackson Laboratory), IFITM3 KO x OT-I.CD45.1, gBT-I.GFP, IFNAR KO, IFNAR x IFNγR KO, IFNγKO, IRF3 KO, IRF7 KO (provided by T. Taniguchi), and IRF3 x IRF7 KO mice were bred in-house and housed in specific pathogen-free conditions in the animal facility at the Peter Doherty Institute of Infection and Immunity, the University of Melbourne. All experiments were approved and performed in accordance with the Institutional Animal Care and Use Committee guidelines of the University of Melbourne. Mice were infected intranasally with X31-OVA [26] (encodes the OVA_257–264_ epitope within the neuraminidase stalk) generously provided by Prof. S. Turner, Monash University, Melbourne, Australia. For total respiratory tract (TRT) infection, mice were anesthetized with inhalation anaesthetic and infected with 10^4^ PFU of X31-OVA in a volume of 30μl.

### Western blot

Total cell lysates were run on a 4–12% SDS/PAGE gel (Invitrogen) and transferred onto nitrocellulose membrane. Membranes were stained with antibodies against mouse IFITM3 (Abcam) and Actin (Sigma). Horseradish peroxidase (HRP)–conjugated anti-rabbit IgG antibody (Sigma) was used for protein detection.

### In vitro activation of T cells

T cells were purified from the LN and spleen of mice. Cells were purified after a depletion step using antibodies against CD11b (M1/70), F4/80, Ter-119, Gr-1 (RB6), MHC class II (M5/114), and CD4 (GK 1.5) or CD8 (YTS 169.4), followed by incubation with anti-rat IgG-coupled magnetic beads (Dynal Biotech) following the manufacturer’s protocols. T cell preparations were 90–95% pure as determined by flow cytometry. Purified naïve CD4^+^ or CD8^+^ T cells were plated at 10^6^ cells per well in 2-ml 24-well flat-bottomed tissue culture plates (Falcon) coated with anti-CD3(145-2C11) and anti-CD28 (37.51). Alternatively, OT-I cells were plated at 10^6^ cells per well in 2-ml 24-well flat-bottomed tissue culture plates (Falcon) with 1 μg/ml SIINFEKL peptide.

### Influenza virus strains

Influenza A virus X31 (H3N2) and Phil82X (H3N2) are high-yielding reassortants of A/Aichi/2/1968 (H3N2) and A/Philippines/2/1982 (H3N2), respectively, with PR8 that express the H3N2 surface glycoproteins. In addition, we have used the seasonal H1N1 influenza virus strains A/Brazil/11/78 (Braz78) and A/New Caledonia/20/1999 (New Cal/99), as well as the H3N2 strains A/Udorn/2/1972 (Udorn72), A/Port Chalmers/1973 (Port Chalmers/73) A/Beijing/353/89 (Beij89). Viruses were grown in 10-day embryonated chicken’s eggs by standard procedures. Titres of infectious virus were determined by plaque assay on Madin-Darby canine kidney (MDCK) cells [27].

### In vitro infection of T cells with influenza virus

Naïve CD4^+^ or CD8^+^ T cells magnetically enriched as described above, or sorted purified from the LN and spleen of mice, were in some experiments labelled with cell trace violet and cultured (10^5^ cells/well) in serum free media with influenza virus (moi = 1–10) for 2 hours. Cells were then washed to remove free virus and cultured at 37°C in RPMI with 10% FCS for 12–48 hours. Susceptibility to infection was measured by intracellular staining for the nucleoprotein (NP) of influenza A viruses using anti-influenza NP-FITC (431, Abcam).

### In vitro activation and adoptive transfer of OT-I

A total of 5 × 10^7^ transgenic splenocytes from OT-I mice were cultured for 4 days with 5 × 10^7^ C57BL/6 splenocytes pulsed with 0.1 μg of OVA_257–264_ peptide, in 40 ml of RPMI 1640 supplemented with 10% FCS, 2 mM glutamine, 5 × 10^−5^ M 2-ME, and antibiotics. The cultures were diluted 1 in 2 on days 2 and 3 with fresh medium containing 10 U/ml IL-2. On day 4, cells were collected, and 5 × 10^6^ T cells were transferred into recipient mice via intravenous (i.v.) injections in a total volume of 200 μl of PBS

### Immunohistochemistry

Perfused lung tissue, inflated with OCT were immediately embedded in OCT. Frozen sections (14 μm) were cut using a cryostat. Tissue sections were fixed in acetone, blocked in serum-free protein block and then stained for CD8 (53–6.7), CD45.1 (A20) and DAPI. Images were acquired on a Zeiss LSM700 microscope with a 20x objective. Acquired images were processed using ImageJ software.

### Flow cytometry and cell sorting

Single cell suspensions were prepared from spleens by mechanical disruption. Mice were perfused prior to the harvest of the lung tissue which was then incubated for 1 h at 37°C in 3 mL of collagenase type 3 (Worthington), (3 mg/mL in RPMI 1640 supplemented with 2% FCS) and DNAse I (0.5mg/ml) (Roche). Cells were stained for 25 min on ice with the appropriate mixture of monoclonal antibodies and washed with PBS with 1% BSA. The conjugated monoclonal antibodies were obtained from BD Pharmingen or Biolegend include; anti-CD8 (53–6.7), anti-CD45-1 (A20), anti-CD45.2(104). H2-D^b^-NP-tetramer was made in house. The levels of IFN-γ, IFN-ß, and interlukin-6 (IL-6) were analysed using a BD Cytometric Bead Array (CBA) Mouse Inflammation Kit (LegendPlex, Biolegend, San Diego, CA, USA) according to the manufacturer’s instructions.

### Statistical analysis

Data were analysed with GraphPad Prism and the indicated statistical tests. * P<0.05, ** P<0.01, *** P<0.001.

## Results and discussion

### Activation-induced up-regulation of IFITM3 on CD8 T cells occurs independently of type I and II interferon

**Fig 1:**
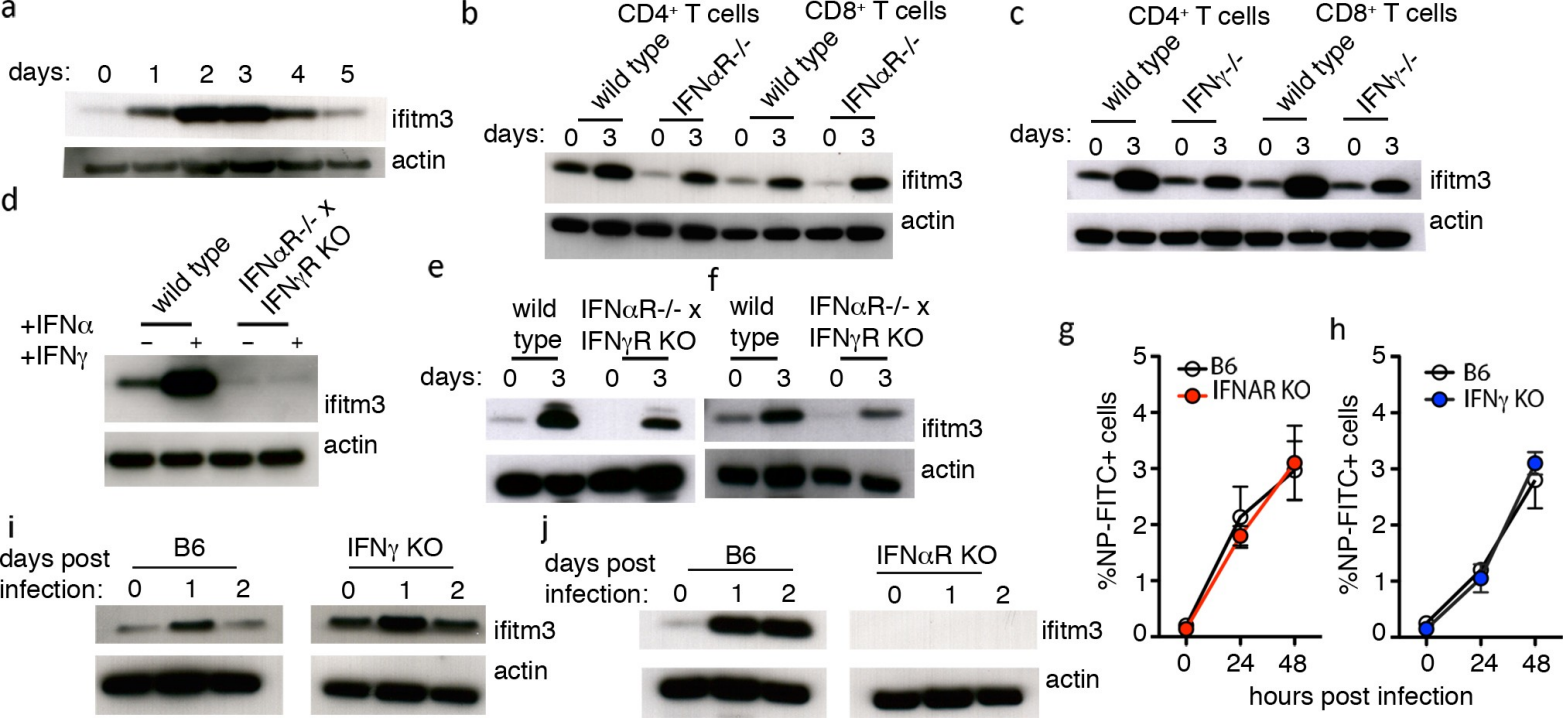
Activation induced up-regulation of IFITM3 on T cells occurs independently of type I and type II interferons. (A) Western blot analysis of IFITM3 expression by CD8^+^ T cells before (0) and after *in vitro* activation with anti-CD3/28 and 1–5 days in culture. Actin was included as a loading control throughout. (B) Western blot analysis of IFITM3 expression by CD4^+^ and CD8^+^ T cells from WT or IFNAR^-/-^ before (0) and 3 days after *in vitro* activation with anti-CD3/28. Data are representative of three independent experiments. (C) Western blot analysis of IFITM3 expression by WT IFNγ ^-/-^ CD4^+^ and CD8^+^ T cells before (0) and 3 days after *in vitro* activation with anti-CD3/28. Data are representative of three independent experiments. (D) Western blot analysis of IFITM3 expression by WT or IFNγR x IFNAR KO CD8^+^ T cells before (-) and 18 hrs after exposure to IFNα and IFNγ. Data are representative of three independent experiments. (E-F) Western blot analysis of IFITM3 expression by WT or IFNγR x IFNAR KO naïve (E) CD4^+^ and (F) CD8^+^ T cells before (0) and 3 days after *in vitro* activation with anti-CD3/28. Data are representative of three independent experiments. (G-H) Intracellular staining for influenza A virus nuclear protein (Flu-NP) in naïve CD8^+^ T cells isolated from WT B6 and (G) IFNAR KO or (H) IFNγ KO mice infected *in vitro* with influenza virus strain X31 (multiplicity of infection, moi = 1) and cultured for 24 and 48 hrs. Data are pooled from 3 independent experiments. Symbols represent the mean ± SEM. (I-J) Western blot analysis of IFITM3 expression by WT B6 CD8^+^ T cells and (I) IFNγ KO CD8^+^ T cells or (J) IFNAR KO CD8^+^ T cells before (0) and 1 and 2 days after infection with influenza virus strain X-31 (moi = 1). Data are pooled from 3 independent experiments.

IFITM3 is classically defined as an interferon stimulated gene (ISG) whose expression is known to be up-regulated in response to type I and II interferons [6]. We have previously demonstrated that naïve CD8^+^ T cells do not express IFITM3 however following crosslinking of the TCR which triggers activation, T-cells transiently upregulate expression of this antiviral protein (Fig 1A, [17]). Here, we investigated if the upregulation of IFITM3 by activated T cells was in response to interferon signalling. To do this, naïve CD4^+^ and CD8^+^ T cells purified from wild type (WT) mice, IFNAR KO mice (which lack the IFNα receptor and therefore are unable to respond to type I interferon), or IFNγ KO mice (which are unable to synthesize interferon gamma), were activated *in vitro* using plate bound anti-CD3/CD28 and 3 days later the expression of IFITM3 in naïve (day 0) and activated T cells was measured (Fig 1B and 1C). Both CD8^+^ and CD4^+^ T cells that lacked the IFNα receptor upregulated IFITM3 expression following TCR engagement (Fig 1B), as did activated T cells that were defective in IFNγ synthesis (Fig 1C). To determine if there was redundancy between the type I and II interferon signalling pathways, and to exclude involvement of either of these cytokines in T cell activation-induced IFITM3 expression, we used mice that lacked both the IFNα and IFNγ receptors. Naïve T cells isolated from these animals failed to express IFITM3 in response to type I and II interferon exposure, confirming that these cells lacked all responsiveness to type I and II interferons (Fig 1D). Following *in vitro* activation of these cells (as described above) we observed upregulation of IFITM3 expression, albeit less effectively (Fig 1E and 1F) indicating that the induction of IFITM3 expression by engagement of the TCR was, at least in part, independent of type I and II interferon signalling.

Interestingly, while IFN signalling was not required for TCR engagement induced up-regulation of IFITM3, it was crucial for evoking IFITM3 expression following exposure to virus. To show this we infected WT or IFNAR KO or IFNγ KO CD8^+^ T cells with influenza virus (X31, H3N2) and cultured these cells *in vitro* for 48 hrs. This infection protocol resulted in roughly 3% of the total WT and KO CD8^+^ T cells being infected with influenza virus, as measured by intracellular staining for the virus nuclear protein (NP) (Fig 1G and 1H). While both WT and IFNγ KO T cells up-regulated IFITM3 expression within 24 hrs of exposure to influenza virus (Fig 1I), T-cells that lacked the ability to respond to type I interferon did not increase IFITM3 expression in response to virus (Fig 1J). Collectively these data demonstrate that distinct signalling pathways drive IFITM3 expression in T cells following TCR engagement or exposure to influenza virus.

### Activation induced up-regulation of IFITM3 on CD8 T cells is not driven by a secreted factor and occurs independently of IRF3 and IRF7

**Fig 2:**
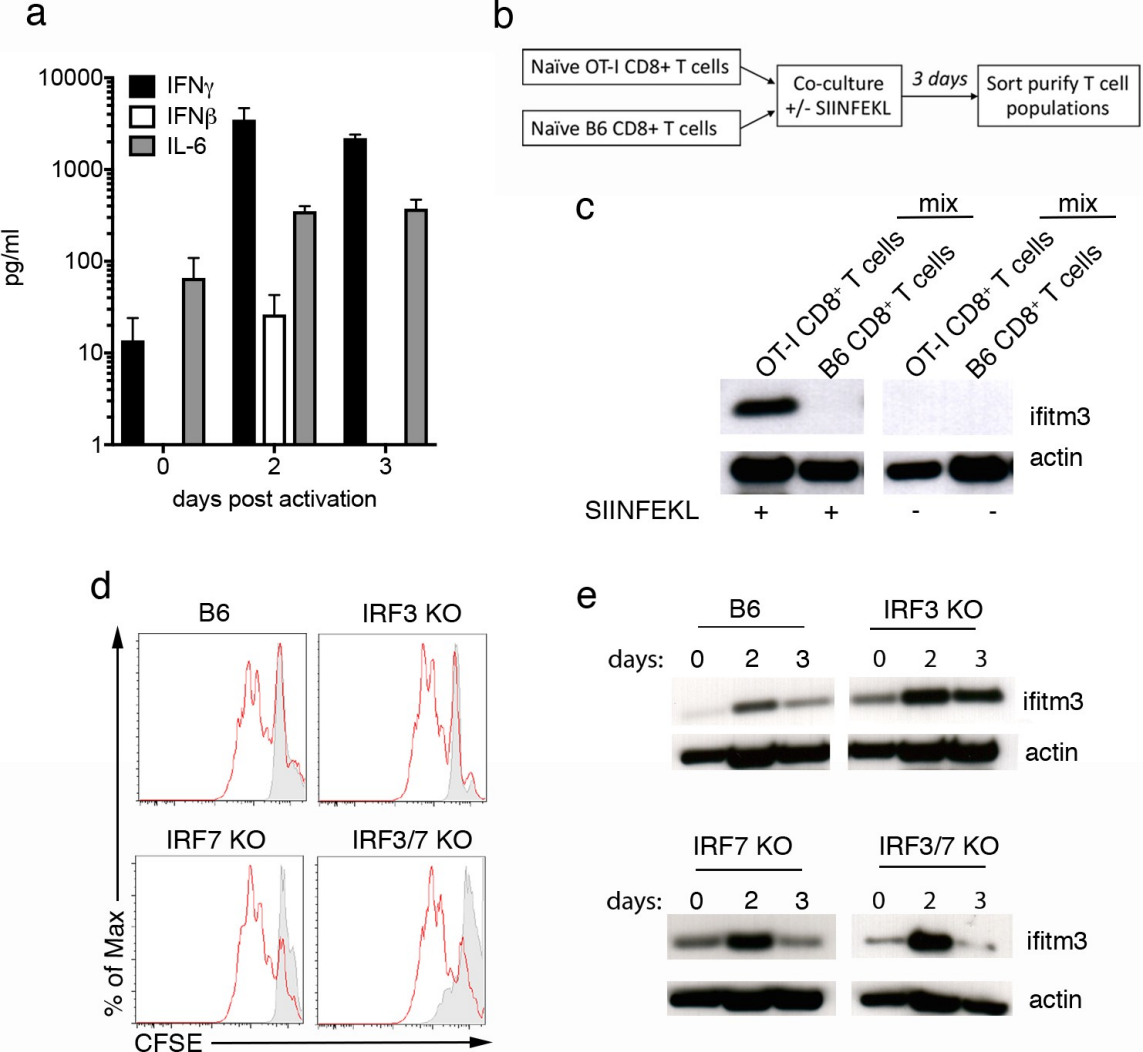
Activation-induced up-regulation of IFITM3 expression by T cells is not driven by a secreted factor and occurs independently of the transcription factors IRF3 and IRF7. (A) Supernatants from CD8^+^ T cell cultures, before (0) and 2–3 days after *in vitro* activation with anti-CD3/28 were recovered and the level of inflammatory cytokines was measured using a cytometric bead array. Data pooled from 3 independent experiments. Bars represent the mean + SEM. (B-C) TCR transgenic OT-I CD8^+^ T cells and B6 CD8^+^ T cells were mixed 1:1 and co-cultured in the presence of SIINFEKL peptide for 3 days. After co-culture, OT-I and B6 cells were sort purified and levels of IFITM3 expression were determined by western blot. (B) Schematic of the experimental procedure. (C) Western blot depicting levels of IFITM3 expression. Data are representative of 3 experiments. Actin was included as a loading control throughout. (D) CFSE-labelled naïve CD8^+^ T cells purified from WT, IRF3 KO, IRF7 KO, or IRF3/7 KO were activated *in vitro* with anti-CD3/28 and cultured for 3 days. Representative flow cytometry profiles of CFSE expression confirm division of CD8^+^ T cells. Grey histograms represent unstimulated cells. (E) Western blot analysis of IFITM3 expression by WT (B6), IRF3 KO, IRF7 KO, or IRF3/7 KO CD8^+^ T cells before (0) and 2–3 days after *in vitro* activation with anti-CD3/28. Data are representative of three independent experiments.

In addition to type I and II interferon, other cytokines, including members of the gp130 family of cytokines (i.e. IL-6) are known inducers of IFITM3 expression [6]. We could detect the presence of both type I and II interferon as well as IL-6 in the supernatant of T cell cultures following TCR engagement (Fig 2A), as such, we investigated if secreted factors released by T cells during their activation were acting in a paracrine manner to induce IFITM3 expression. To do this we purified ovalbumin (OVA)-specific CD8^+^ T cells from OT-I mice and co-cultured these cells with naïve polyclonal CD8^+^ T cells from B6 mice. The SIINFEKL peptide was added to the culture to activate only the transgenic OT-I T cells and on day 3 post co-culture, activated OT-I cells were separated from naïve B6 bystander CD8^+^ T cells, and IFITM3 expression was determined in each of the T cell populations (Fig 2B). Consistent with our prior studies [17], following exposure to their cognate antigen (SIINFEKL), OT-I cells upregulated IFITM3 expression (Fig 2C). In contrast, naïve B6 bystander CD8^+^ T cells isolated from the same well, and therefore exposed to the same cytokine milieu, did not upregulate IFITM3, suggesting that a secreted factor was not driving activation induced up-regulation of IFITM3 by CD8^+^ T cells (Fig 2C).

Interferon-induced up-regulation of IFITM3 has previously been shown to be driven by the transcription factors interferon regulatory factor 3 and 7 (IRF3 and IRF7) [28]. We next determined if these transcription factors also regulated expression of IFITM3 following TCR engagement. We first confirmed that CD8^+^ T cells isolated from mice deficient in either IRF3 or IRF7, or both, showed equivalent levels of activation and expansion in response to TCR crosslinking. To do this, naïve CD8^+^ T cells purified from either WT, IRF3 KO, IRF7 KO or IRF3 x IRF7 KO mice were labelled with carboxyfluorescein succinimidyl ester (CFSE) and activated *in vitro* with plate bound anti-CD3/28 for 3–5 days. We observed similar proliferation profiles (Fig 2D) and could recover similar numbers of divided cells (S1 Fig) from the cultures of WT and KO CD8^+^ T cells, confirming that deficiencies in these transcription factors does not impact T cell activation. To determine if IRF3 or IRF7 are involved in TCR engagement-induced IFITM3 expression, we purified naïve CD8^+^ T cells from WT and KO mice, activated these cells as described above and, on days 2 and 3 post-activation, measured IFITM3 expression. CD8^+^ T cells deficient in IRF3, IRF7 or both IRF3 and IRF7 upregulated IFITM3 following TCR engagement, indicating that these transcription factors are not required for activation-induced IFITM3 expression (Fig 2E).

### Division of CD8^+^ T cells is associated with upregulation of IFITM3 expression and enhanced resistance to influenza virus infection

**Fig 3:**
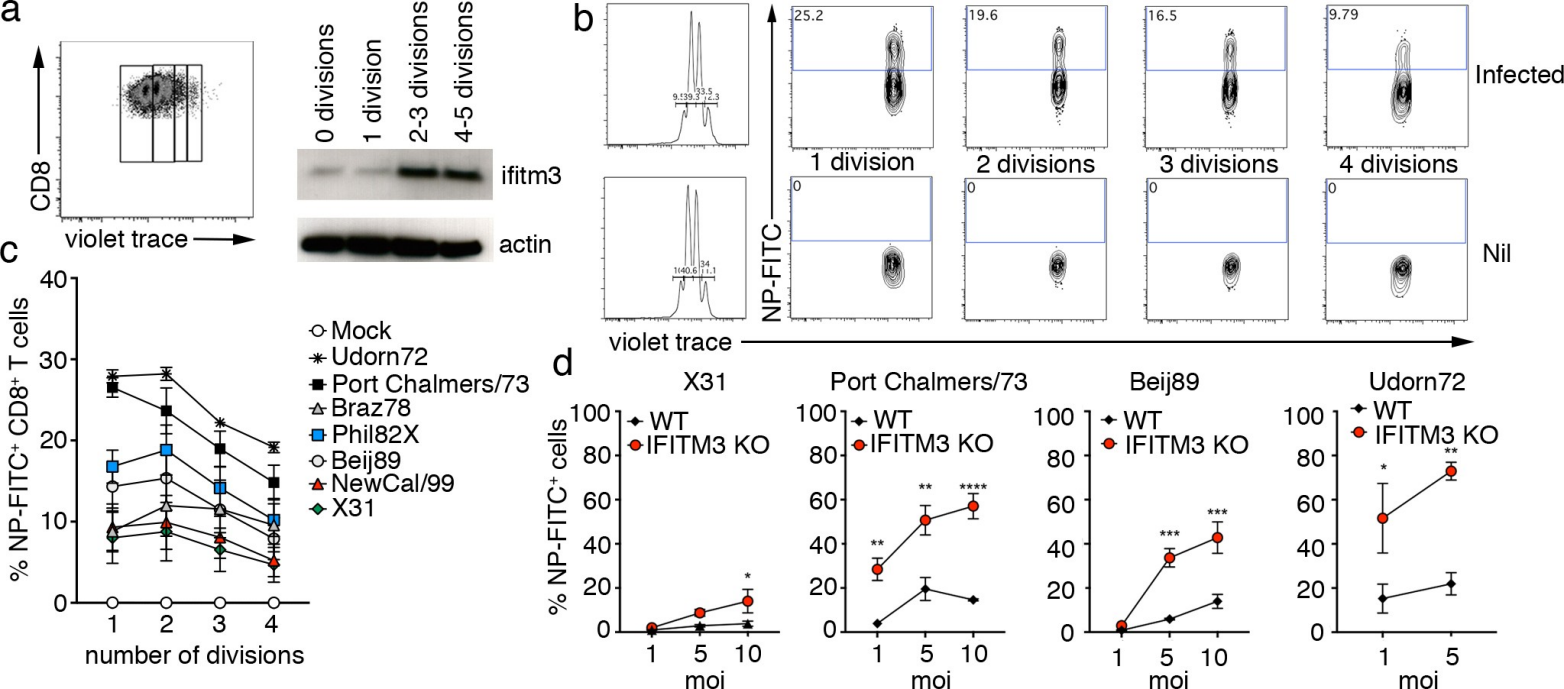
Division of CD8^+^ T cells is associated with upregulation of IFITM3 expression and enhanced resistance to influenza virus infection. (A) Naïve OT-I CD8^+^ T cells were labelled with cell trace violet, activated *in vitro* with SIINFEKL peptide for 3 days and then sorted into subsets of cells undergoing 0–5 division based on dilution of the membrane dye. Western blot expression levels of IFITM3 by cells undergoing 0, 1–2, 3–4, >5 divisions. Data are representative of 2 experiments. Actin was included as a loading control throughout (B) Naïve OT-I CD8^+^ T cells labelled with cell trace violet and activated *in vitro* with SIINFEKL peptide for 3 days were infected with different strains of influenza A viruses (moi = 10), and 12 hrs later the proportion of infected cells at each cell division was measured by intracellular staining for influenza A virus nucleoprotein (Flu-NP). Representative flow cytometry profiles are shown. (C) Percentage of influenza virus-infected (Flu-NP^+^) cells at each cellular division. Data are pooled from 3 independent experiments. (D) IFITM3 KO OT-I or WT OT-I were activated *in vitro* with SIINFEKL peptide for 3 days and infected with different strains of influenza A virus (moi = 1, 5 and 10, as indicated) and 12 hrs later the percentage of virus-infected cells was measured by intracellular staining for influenza A virus nucleoprotein (Flu-NP). Data are pooled from 2 independent experiments (two way ANOVA, Sidak’s multiple comparison).

To gain insight into the kinetics of TCR engagement-induced expression of IFITM3, we labelled naïve B6 CD8^+^ T cells with violet trace membrane dye, activated these cells *in vitro* with plate-bound anti-CD3/28 for 3 days and then sort purified T cells into 4 groups according to the number of divisions the cells had undergone (i.e. 0 divisions, 1 division, 2–3 divisions, 4–5 divisions). We assessed each group of cells for expression of IFITM3 and found that only after cells had undergone 2–3 rounds of division could upregulation of IFITM3 be detected (Fig 3A). We next assessed if the expression of IFITM3 on dividing T-cells increased their resistance to influenza virus infection. To do this, violet trace dye-labelled naïve CD8^+^ T cells were activated *in vitro* (as described above) for 3 days, infected with a panel of different influenza A virus strains, and 12 hrs later the proportion of infected cells (Flu-NP+) undergoing 1–4 divisions was determined (Fig 3B and 3C). While different influenza A virus strains showed striking differences in their ability to infect CD8^+^ T cells (with infection rates ranging from 5–28%) in general, we observed less infection of T cells that had undergone 4 rounds of division compared to T cells that had undergone 1–2 rounds of division. Thus, as T cells undergo division, expression of the antiviral IFITM3 protein increases, as does resistance to influenza virus infection.

To directly determine if the enhanced resistance of activated CD8^+^ T cells to influenza A virus infection was mediated by IFITM3, transgenic WT OT-I CD8^+^ T cells and IFITM3 KO OT-I CD8^+^ T cells were activated *in vitro* for 3 days and then infected with different influenza A viruses (multiplicity of infection, moi = 1, 5, 10) and, 12 hrs later, the percentage of virus-infected cells was determined (Fig 3D). For the four strains of influenza A virus tested, activated T cells deficient in IFITM3 expression were infected to significantly higher levels, indicating that IFITM3 expression can protect effector T cells from influenza virus infection (Fig 3D).

### IFITM3 protects effector T cells from influenza virus infection and imparts a survival advantage at sites of virus infection

**Fig 4:**
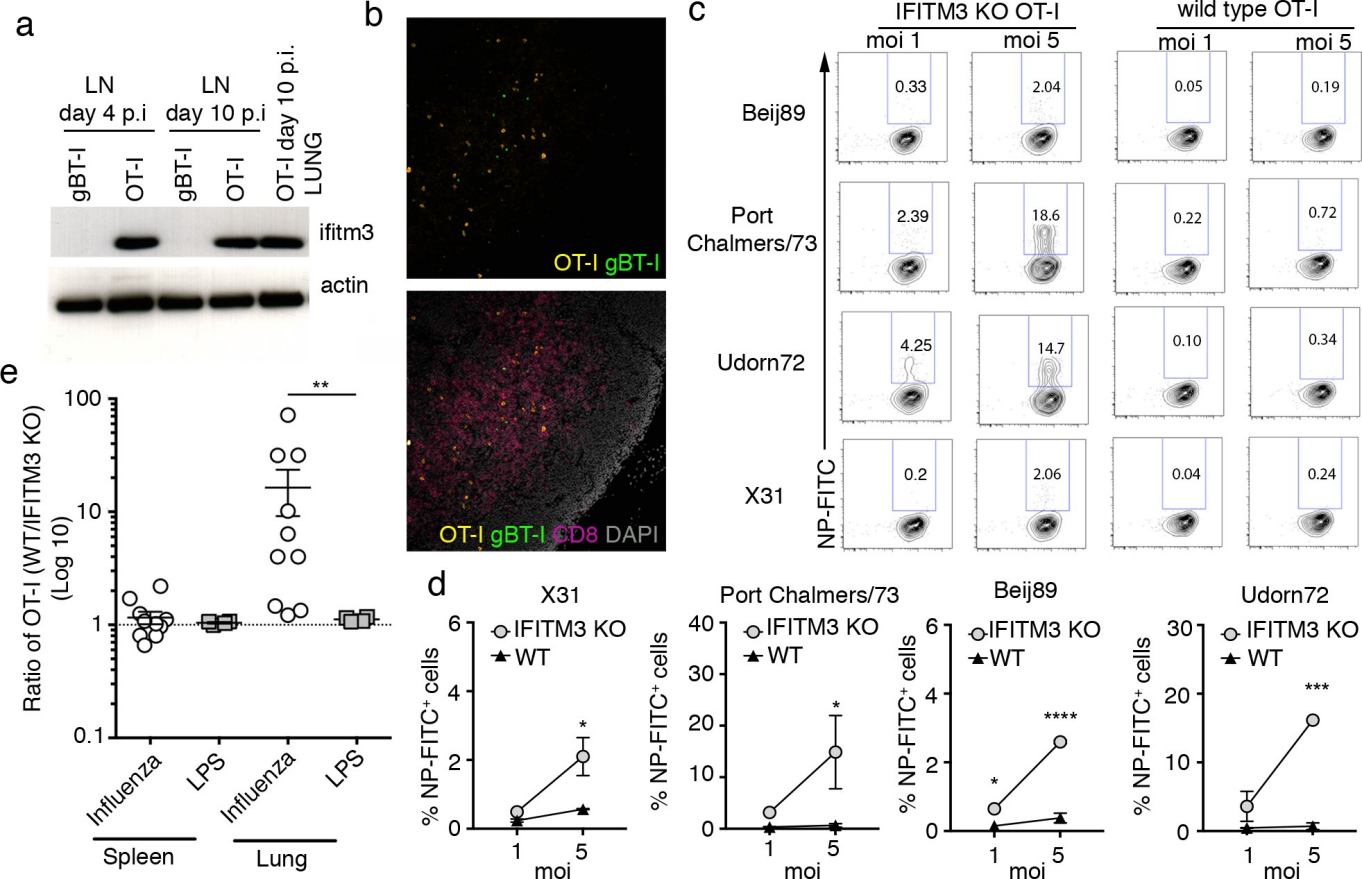
Activated CD8^+^ T cells up-regulate IFITM3 in vivo during influenza virus infection and this confers a survival advantage at the site of infection. (A) Mice seeded with naïve gBT-I.GFP^+^ and naïve OT-I.CD45.1 CD8^+^ T cells prior to i.n. infection with 10^4^ PFU of X31-OVA. On day 4 and 10 p.i., OT-I and gBT-I were sort purified from the lung and the lung-draining LN and levels of IFITM3 expression were determined by western blot. Data are representative of 2 experiments. Actin was included as a loading control. (B) Histology on the LN at day 4 p.i. showing staining for CD8 (pink), gBT-I.GFP^+^(green), OT-I.CD45.1(yellow) and DAPI (grey) (C) Mice seeded with 10^4^ naïve IFITM3 KO OT-I or WT OT-I.CD45.1 CD8^+^ T cells and infected i.n. with 10^4^ PFU X31-OVA. On day 7 p.i., WT and IFITM3 KO effector OT-I were sort purified from the spleen and LN and infected *in vitro* with different influenza A viruses (moi = 1 or 5) and 12 hrs later the proportion of influenza virus-infected cells was measured by intracellular staining for influenza A virus nucleoprotein (Flu-NP). Representative flow cytometry profiles are shown. (D) Percentage of influenza virus-infected (Flu-NP+) cells. Data are pooled from 2 experiments, symbols represent the mean ± SEM. (two-way ANOVA, Sidak’s multiple comparison) (E) Mice were infected i.n. with X31-OVA or treated i.n. with LPS and 2 days later received 5 x 10^6^ *in vitro*-activated WT and IFITM3 KO OT-I T cells. The ratio of WT to IFITM3 KO OT-I T cells in the spleen and lung was then determined 48 hrs later. Data are pooled from 3 independent experiments, dots represent individual mice (n = 4–10, Mann-Whitney test).

We next investigated if IFITM3 expression was upregulated by effector T cells *in vivo* during influenza virus infection and whether this impacted their ability to mediate their effector functions. First, we tracked the kinetics of IFITM3 expression by T cells activated in mice during an influenza virus infection. To do this, C57BL/6 mice (CD45.2) were seeded with congenically marked (CD45.1) OT-I CD8^+^ T cells and, as a control, green fluorescent protein (GFP)-expressing TCR transgenic CD8^+^ T cell specific for the glycoprotein B of herpes simplex virus (gBT-I), prior to intranasal infection with a recombinant influenza virus expressing the model antigen OVA (X31-OVA). On days 4 and 10 post infection (p.i.), both transgenic CD8^+^ T cell populations were sort purified from the lung-draining lymph node (LN) and levels of IFITM3 expression was determined. At both 4 and 10 days p.i., effector OT-I T cells isolated from the LN of X31-OVA-infected mice expressed IFITM3 while the naïve gBT-I T cells did not (Fig 4A). Immunofluorescence analysis of the LN on day 4 p.i. revealed that both transgenic T cells were in the similar locations, confirming that merely being in the same inflammatory environment is not sufficient for naive T cells to upregulate IFITM3 expression (Fig 4B). The expression pattern of IFITM3 we observed was not unique to the transgenic T-cells as endogenous naïve (CD44^-^) and activated (CD44^+^) CD8^+^ T cells recovered from lung draining LN on day 4 and 10 p.i. displayed a similar pattern of IFITM3 expression (S2 Fig). Thus, only T cells within the LN that had undergone cognate antigen recognition upregulated IFITM3 *in vivo*. Effector OT-I cells recovered from the lung on day 10 p.i also expressed IFITM3 suggesting that activated T cells retain expression even after they exit the LN and infiltrate infected tissue (Fig 4A). To confirm that IFITM3 expression by *in vivo*-activated CD8^+^ T cells was associated with increased resistance of these cells to influenza virus infection, mice seeded with WT and IFITM3 KO OT-I cells were infected intranasally within X31-OVA and, on day 7 p.i., both OT-I cell populations were purified from the LN and spleen and infected *in vitro* with a panel of influenza viruses. Infection efficiency, assessed as the percentage of influenza A virus NP^+^ cells, was measured 12 hrs later. Consistent with results obtained using *in vitro*-activated CD8^+^ T cells (see Fig 3D), effector OT-I cells activated *in vivo* which lacked IFITM3 expression were also more susceptible to influenza virus infection compared to WT effector OT-I cells (Fig 4C and 4D). We next assessed if the pattern of IFITM3 expression and corresponding enhanced resistance to influenza virus infection was also a feature of endogenous influenza specific CD8^+^ T cells. To do this we infected WT and IFITM3 KO mice with influenza virus (X31) and on day 7 p.i. influenza virus nuclear protein (NP) specific CD8^+^ T cells (NP-tetramer^+^) were sort purified from the lung draining LN and spleen (S3A Fig). We confirmed that WT NP-tetramer^+^ CD8^+^ T cells expressed elevated levels of IFITM3 (S3B Fig) and then tested if this IFITM3 expression increased the resistance of these influenza specific CD8^+^ T cells to virus infection. To do this, we infected *in vitro* either WT or IFITM3 KO NP-tetramer^+^ CD8^+^ T cells with a panel of influenza viruses and measured the proportion of infected cells 12 hrs later by staining for the expression of viral nuclear protein. Once again consistent with results obtained using *in vitro*-activated CD8^+^ T cells (see Fig 3D) and effector OT-I cells activated *in vivo* (see Fig 4C and 4D), we find endogenous *in vivo* activated influenza NP-specific CD8^+^ T cells which lacked IFITM3 expression were also more susceptible to influenza virus infection compared to NP-specific CD8^+^ T cells that could up-regulate this antiviral protein (S3C Fig).

Finally, we determined if the up-regulation of IFITM3 on effector T cells provided a survival advantage by rendering them resistant to virus infection. To assess this, equal numbers of *in vitro*-activated WT and IFITM3 KO OT-I cells were transferred into mice that had been infected with influenza virus 2 days prior or, as a control, received intranasal treatment with an inflammatory agent (LPS) and the ratio and absolute numbers of WT and KO OT-I cells in the spleen and lung was measured 48 hrs later. Equivalent proportions of WT and IFITM3 KO OT-I cells were observed in the spleen and in the lung following transfer into mice that received the inflammatory agent LPS, indicating that both WT and KO OT-I cells can infiltrate these tissues effectively (Fig 4E, S4 Fig). In contrast, while we observed an equivalent ratio of WT and IFITM3 KO OT-I cells in the spleen of mice infected with influenza virus, we observed 10-fold more WT OT-I cells in the lung, suggesting that IFITM3 KO OT-I are at a disadvantage at the site of virus infection (Fig 4E, S4 Fig). Overall, our experiments show that expression of IFITM3 on effector T cells infiltrating the site of virus infection provided these cells a survival advantage allowing them to mediate their effector functions without becoming a target for infection.

The pharmacological induction of IFITM3 could represent a broad-spectrum therapeutic treatment for a range of pathogenic agents. Although injection of type I IFN can induce IFITM3 and other antiviral responses, undesirable off target effects associated with this type of therapy limit its current clinical use. More refined approaches triggering the specific induction of antiviral proteins independently of IFN will provide antiviral resistance with minimal side effects. Here we show that IFITM3 can be up-regulated on T cells independent of IFN signalling. If defined and harnessed, this non-IFN pathway of IFITM3 induction could be targeted as an antiviral therapeutic approach providing the coveted antiviral actions of IFITM3 while avoiding the undesirable, pleiotropic effects of interferon treatment.

## Supporting information

S1 Fig Deficiencies in IRF3 or IRF7 transcription factors does not impact T cell activation. CFSE-labelled naïve CD8^+^ T cells purified from WT, IRF3 KO, IRF7 KO, or IRF3/7 KO were activated *in vitro* with anti-CD3/28 and the absolute number of divided cells was measured 5 days later. Data pooled from 3 independent experiments. Graph shows the mean ± SEM.
(PDF)

S2 Fig Activated CD8^+^ T cells up-regulate IFITM3 in vivo during influenza virus infection. Western blot analysis of IFITM3 expression by endogenous (endo) naïve (CD44^-^) and activated (CD44^+^) CD8^+^ T cells recovered from the LN of mice on day 4 and 10 p.i. with 10^4^ PFU of X31-OVA. Data are representative of 2 experiments. Actin was included as a loading control.
(PDF)

S3 Fig Activated influenza specific CD8^+^ T cells up-regulate IFITM3 in vivo during influenza virus infection and this increases their resistance to influenza virus infection. (A-B) Mice, WT or IFITM3 KO were infected with 10^4^ PFU of X31 and on day 7 p.i influenza specific (NP-tetramer^+^) cells were sort purified from the lung draining LN. (A) Representative flow cytometry profiles depicting the gating strategy for sorting the NP-tetramer+ cells. (B) Western blot analysis of IFITM3 expression by endogenous naïve (CD44^-^) and NP-tetramer^+^ CD8^+^ T cells recovered from the LN of WT mice on day 7 p.i. Data are representative of 2 experiments. Actin was included as a loading control. (C) WT and IFITM3 KO NP-tetramer^+^ cells sort purified from the spleen and LN and infected *in vitro* with different influenza A viruses (moi = 5) and 12 hrs later the absolute number of influenza virus-infected cells was measured by intracellular staining for influenza A virus nucleoprotein (NP-FITC). Data are pooled from 2 experiments, bars represent the mean ± SEM.
(PDF)

S4 Fig Activated CD8^+^ T cells up-regulate IFITM3 in vivo during influenza virus infection and this confers a survival advantage at the site of infection. Mice were infected i.n. with X31-OVA (Influenza) or treated i.n. with LPS and 2 days later received 5 x 10^6^ *in vitro* activated WT and IFITM3 KO OT-I T cells. The absolute number of WT and IFITM3 KO OT-I T cells in the (A) spleen and (B) lung was then determined 48 hrs later. Data are pooled from 3 independent experiments, dots represent individual mice.
(PDF)
