# Supplementary figures and images for: Rapid interferon independent expression of IFITM3 following T cell activation protects cells from influenza virus infection
Source: PLoS One. 2019 Jan 16;14(1):e0210132. doi: 10.1371/journal.pone.0210132 (PMC6334895; doi:10.1371/journal.pone.0210132)

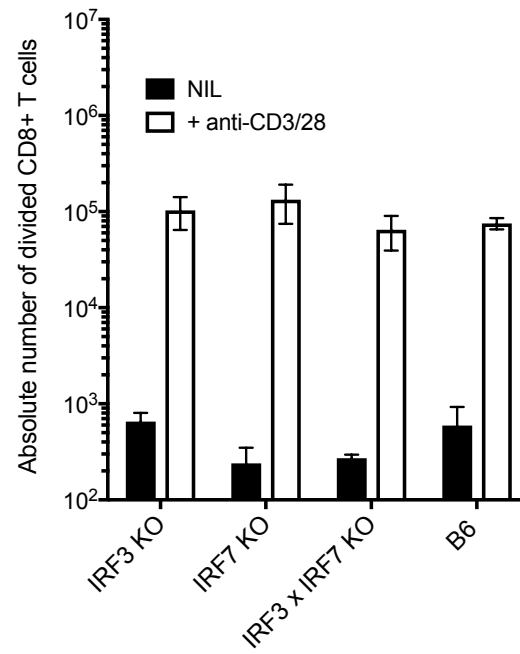

**S1 Fig. Deficiencies in IRF3 or IRF7 transcription factors does not impact T cell activation**

Supplement: S1 Fig — CFSE-labelled naïve CD8+ T cells purified from WT, IRF3 KO, IRF7 KO, or IRF3/7 KO were activated in vitro with anti-CD3/28 and the absolute number of divided cells was measured 5 days later. Data pooled from 3 independent experiments. Graph shows the mean ± SEM. (PDF) [file pone.0210132.s001.pdf]

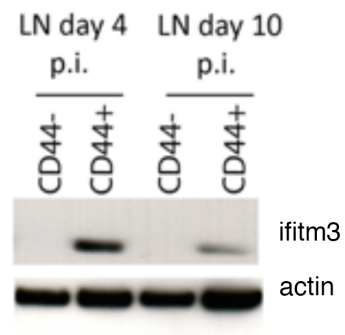

**S2 Fig. Activated CD8<sup>+</sup> T cells up-regulate IFITM3 in vivo during influenza virus infection**

Supplement: S2 Fig — Western blot analysis of IFITM3 expression by endogenous (endo) naïve (CD44-) and activated (CD44+) CD8+ T cells recovered from the LN of mice on day 4 and 10 p.i. with 104 PFU of X31-OVA. Data are representative of 2 experiments. Actin was included as a loading control. (PDF) [file pone.0210132.s002.pdf]
