# Supplementary material for: Rapid interferon independent expression of IFITM3 following T cell activation protects cells from influenza virus infection
Source: PLoS One. 2019 Jan 16;14(1):e0210132. doi: 10.1371/journal.pone.0210132 (PMC6334895; doi:10.1371/journal.pone.0210132)
Supplement: S3 Fig — (A-B) Mice, WT or IFITM3 KO were infected with 104 PFU of X31 and on day 7 p.i influenza specific (NP-tetramer+) cells were sort purified from the lung draining LN. (A) Representative flow cytometry profiles depicting the gating strategy for sorting the NP-tetramer+ cells. (B) Western blot analysis of IFITM3 expression by endogenous naïve (CD44-) and NP-tetramer+ CD8+ T cells recovered from the LN of WT mice on day 7 p.i. Data are representative of 2 experiments. Actin was included as a loading control. (C) WT and IFITM3 KO NP-tetramer+ cells sort purified from the spleen and LN and infected in vitro with different influenza A viruses (moi = 5) and 12 hrs later the absolute number of influenza virus-infected cells was measured by intracellular staining for influenza A virus nucleoprotein (NP-FITC). Data are pooled from 2 experiments, bars represent the mean ± SEM. (PDF) [file pone.0210132.s003.pdf]

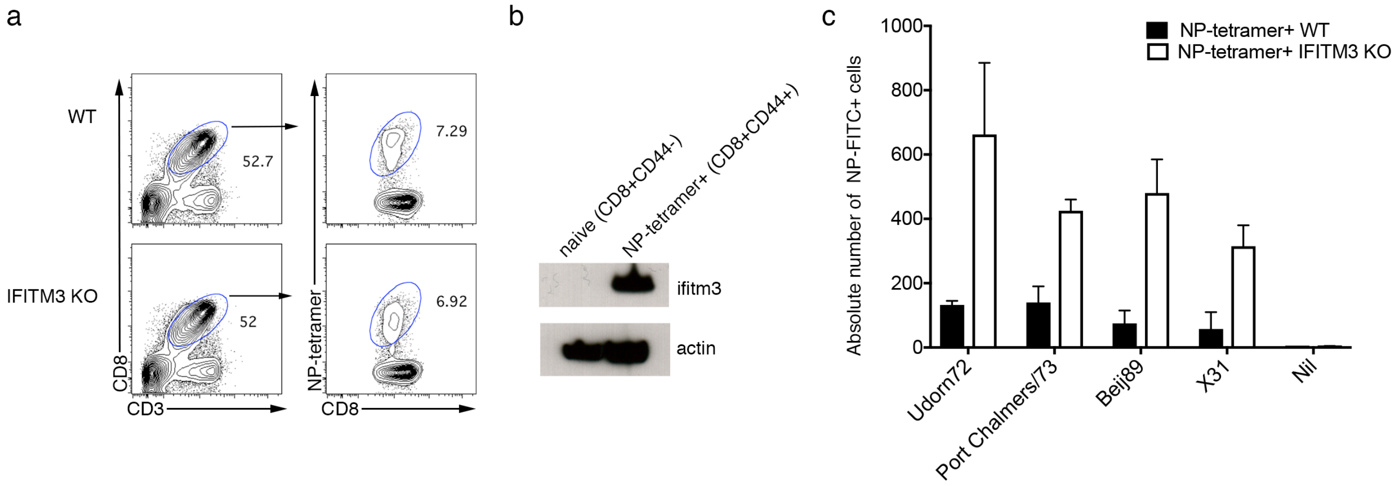

S3 Fig. Activated influenza specific CD8^{+} T cells up-regulate IFITM3 in vivo during influenza virus infection and this increases their resistance to influenza virus infection
