# Supplementary material for: Rapid interferon independent expression of IFITM3 following T cell activation protects cells from influenza virus infection
Source: PLoS One. 2019 Jan 16;14(1):e0210132. doi: 10.1371/journal.pone.0210132 (PMC6334895; doi:10.1371/journal.pone.0210132)
Supplement: S4 Fig — Mice were infected i.n. with X31-OVA (Influenza) or treated i.n. with LPS and 2 days later received 5 x 10^6^ in vitro activated WT and IFITM3 KO OT-I T cells. The absolute number of WT and IFITM3 KO OT-I T cells in the (A) spleen and (B) lung was then determined 48 hrs later. Data are pooled from 3 independent experiments, dots represent individual mice. (PDF) [file pone.0210132.s004.pdf]

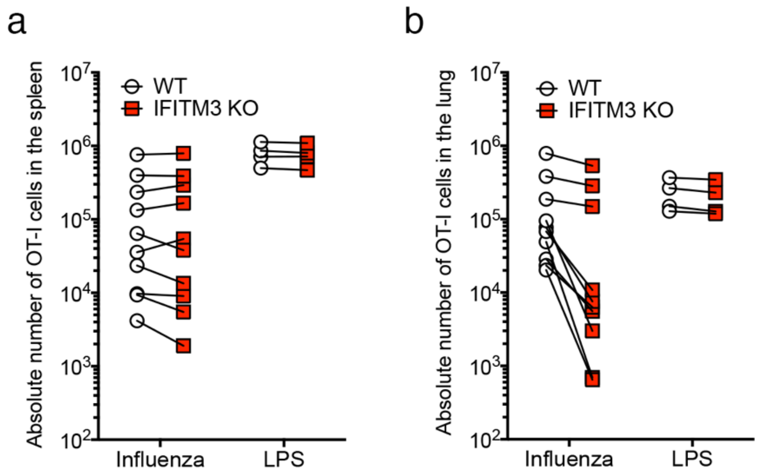

S4 Fig. Activated CD8$^+$ T cells up-regulate IFITM3 in vivo during influenza virus infection and this confers a survival advantage at the site of infection
